# *Weizmannia coagulans* Long45 Supplementation Prevents Feline-Derived *Shigella flexneri 13*-Induced Colitis in Mice by Regulation the Nrf2 and NF-κB Signaling Pathways

**DOI:** 10.3390/nu18101486

**Published:** 2026-05-07

**Authors:** Xinyu Zhang, Yuhe Ma, Haozhen Liu, Yang Yang, Yao Ge, Yinfeng Chen, Ying Yang, Jun Lu, Zhenlong Wu

**Affiliations:** 1 State Key Laboratory of Animal Nutrition and Feeding, College of Animal Science and Technology, China Agricultural University, Beijing 100193, China; 2 Beijing Advanced Innovation Centre for Food Nutrition and Human Health, China Agricultural University, Beijing 100193, China; 3 Auckland Bioengineering Institute, University of Auckland, Auckland 1142, New Zealand; jun.lu@auckland.ac.nz; 4 Department of Food and Agriculture Technology, Yangtze Delta Region Institute of Tsinghua University, Hangzhou 314006, China

**Keywords:** cat, diarrhea, *Shigella*, *Weizmannia coagulans*

## Abstract

**Background:** Shigellosis is an illness that affects young children all over the world and *Shigella flexneri* is one of the most common pathogens. The aim of this study was to investigate a potential beneficial effect of *Weizmannia coagulans* Long45 supplementation on feline-derived *Shigella flexneri*-induced colitis in mice, as well as potential mechanisms. **Results:** The results revealed that mice receiving fecal microbiota from diarrheic cats experienced significant weight loss, decreased survival rate, increased mRNA levels of inflammatory cytokines (TNF-α, IL-4, IFN-γ, IL-1β, IL-6, and IL-18), and increased cell apoptosis compared to the single DSS treatment. In contrast, mice that received fecal microbiota from healthy cats exhibited an increased body weight, increased mRNA level of ZO-1, claudin-3, and Muc2 and decreased apoptosis, indicating a protective effect. The 16S rDNA analysis revealed that the abundance of *Shigella* in the feces of diarrheic cats was significantly higher than that in healthy cats, while the abundance of *Bacillus* was lower. Using bacteria culture technology, 19 strains of *Shigella flexneri* were isolated from 27 fecal samples of diarrheic cats and a strain of *Weizmannia coagulans Long45* was isolated from the feces of healthy cats. Further study showed that *Weizmannia coagulans* Long45 significantly alleviated pathological alterations and colonic barrier dysfunction by modulating the NF-κB and Nrf2 signaling pathways. **Conclusions:** Our data indicate that feline-derived *Shigella flexneri* may be a potential pathogen associated with diarrhea and intestinal barrier dysfunction. *Weizmannia coagulans* Long45, as a potential probiotic, can effectively alleviate *Shigella*-induced colitis by interfering with the Nrf2 and NF-κB signaling pathways.

## 1. Introduction

Shigellosis, also known as bacillary dysentery, is a gastrointestinal disease that affects both humans and animals all over the world [1,2]. Shigellosis is caused by the bacteria of the genus *Shigella*, including *Shigella dysenteriae*, *Shigella flexneri*, *Shigella boydii*, and *Shigella sonnei*, with *S. flexneri* being the most common in developing countries, causing approximately 165 million cases of diarrhea and resulting in up to 1 million deaths annually [3,4]. It has been reported that young children and elderly individuals are vulnerable to *S. flexneri* infection due to weakened immunity and intestinal dysfunction, as well as various stimuli [5,6]. In response to bacterial invasion, a host inflammation response is activated to maintain intestinal homeostasis, resulting in fever, abdominal pain and diarrhea, and affecting individuals’ health, welfare and life quality [7,8]. Antibiotics, such as ciprofloxacin, azithromycin and ceftriaxone, are first-line agents used to relieve symptoms and complications related to shigellosis [9,10]. Notably, *Shigella* spp. have demonstrated resistance to multiple third-generation antibiotics. This alarming trend underscores the urgent need for multifaceted therapeutic strategies [11,12,13]. In addition to humans, *Shigella* infections have been reported in companion animals, such as dogs and cats, after the ingestion of contaminated food or water [14,15]. However, it remains unknown whether feline-derived *S. flexneri* can induce or aggravate colitis and contribute to a deleterious damaging effect in humans and experimental animals.

The gastrointestinal tract harbors hundreds of billions of microorganisms, which interacts with host cells and functions as an essential part of the intestinal barrier. Among these microorganisms, probiotics are the main bacteria that mediate the beneficial effect through multiple mechanisms [16]. *Weizmannia coagulans* (formerly known as *Bacillus coagulans*) is a spore-forming and lactic acid-producing bacterium with an ability to improve gut health by regulating the digestion and metabolism of nutrients, modulating the gut microbiota, and improving host cell immunity [17,18,19]. In our recent study, we identified a strain of *Weizmannia coagulans* (*W. coagulans* Long45) in the colon of an adult cat (unpublished data). It remains unknown whether *W. coagulans* Long45 can reduce *S. flexneri*-induced colitis. In the present study, fecal microbiota transplantation (FMT) technology was used to transplant the fecal microbiota of cats into antibiotic-cocktail-treated mice to assess its effect on colitis-related intestinal barrier dysfunction and inflammation. We found that *S. flexneri* is a predominate pathogen responsible for exacerbated colonic injuries following microbiota transplantation from diseased cats. Additionally, we evaluated a beneficial effect of *Weizmannia coagulans* Long45 supplementation on *S. flexneri*-induced colitis in mice. The data provided herein reveal that feline-derived *Shigella flexneri* may be a potential pathogen associated with diarrhea and intestinal barrier dysfunction. *Weizmannia coagulans Long45*, as a potential probiotic, can effectively alleviate *Shigella*-induced colitis by interfering with Nrf2 and NF-κB signaling.

## 2. Materials and Methods

### 2.1. Fecal Sample Collection

Fecal samples were collected between June 2023 and December 2025. Twenty-seven cats with diarrhea and 10 healthy cats were included in this study from one of the Veterinary Teaching Hospitals in Beijing. All the cats were inoculated with vaccines (Pfizer Purevax Feline Pentavalent Vaccine) and the pet parents were informed of the process. Fresh feces were filtered, centrifuged, stored in 30% glycerol, and placed in a −80 °C refrigerator for later fecal microbiota transplantation (FMT). Laboratory testing was examined and the results for the diarrhea-related virus and parasite were negative.

### 2.2. Fecal Microbiota Transplantation

All experimental protocols in this study were approved by the Animal Care and Use Committee of China Agricultural University (AW32504202-1-1). Six-week-old male C57BL/6 mice (purchased from Beijing HuafuKang Biological Technology Co., Ltd.; Beijing; China) were fed in animal cages with controlled temperature and humidity on a 12 h light–dark cycle with free access to food and water. After one week of adaptation, all the mice were treated with an antibiotic mixture (Penicillin 1 g/L, Streptomycin 1 g/L, Neomycin Sulfate 1 g/L, and Metronidazole 0.5 g/L, administered in drinking water), according to the method previously described [20,21]. Then, all the mice were grouped according to the experiment requirements, with each group having no less than 8 mice. After the experiment was over, the mice were euthanized with carbon dioxide and tissue samples were collected and stored at −80 degrees for subsequent testing and analysis.

### 2.3. Experimental Design

In the fecal microbiota transplantation trial, six-week-old C57BL/6J mice treated with a cocktail of four antibiotics were randomly divided into three groups, which were orally administered with normal saline (Con), a fecal microbiota suspension from healthy cats (FN), or a fecal microbiota suspension from diarrheic cats (FD), respectively. During the initial five days of the experiment, the mice were provided with drinking water that contained 2% DSS on a daily basis. Subsequently, microbial transplantation was performed on days 1, 3, 5, 7, and 9. Finally, on day 10, the mice were euthanized, and related samples were collected for further analysis.

To evaluate the potential efficacy of *Weizmannia coagulans* Long45, six-week-old C57BL/6J mice treated with a cocktail of four antibiotics were randomly divided into four groups. From days 1 to 7, mice in the Con and W.C groups were administered Tryptic Soy Broth (TSB) medium via gavage and mice in the S.F and W.C+ S.F groups were given *Shigella flexneri 13* (2 × 10^7^ CFU/mL, 200 μL/d). From day 8 to 23, mice in the Con and S.F groups continued to receive TSB medium, whereas mice in the W.C and W.C+ S.F groups were administered *Weizmannia coagulans* Long45 (1.73 × 10^9^ CFU/mL, 200 μL/d).

### 2.4. Histopathological Analysis

The colon tissues were fixed in 4% paraformaldehyde for 24 h, and then were stored in 70% alcohol. The colon tissues were sliced by using a rotary microtome (Leica, Wetzlar, Germany) after being dehydrated and paraffin-embed. After dewaxing, colon slices were stained with hematoxylin–eosin (H&E) and Alcian blue/periodic acid–Schiff (AB-PAS). The intestinal morphology was analyzed by using a microscope (Nikon, Tokyo, Japan) and images were taken.

### 2.5. Determination of Biochemical Indicators in Colon

The hydrogen peroxide (H_2_O_2_), catalase (CAT), malondialdehyde (MDA), glutathione S-transferase (GSH-ST) and myeloperoxidase (MPO) in the serum were detected by commercial kits (Nanjing Jiancheng Bioengineering Institute, Nanjing, China) according to the manufacturer’s instructions.

### 2.6. Western Blotting

Colon tissue was lysed with RIPA, followed by centrifugation at 12,000 rpm for 15 min at 4 °C to extract the protein. The extracted protein was separated by SDS-PAGE and transferred into a polyvinylidene fluoride membrane (Millipore, Billerica, MA, USA) later. The membrane was blocked with 5% bovine serum albumin solution for 1 h at room temperature. Subsequently, the membrane was probed with target antibodies over 12 h at 4 °C. Protein bands were visualized by the Image Quant LAS 4000 mini system (GE Healthcare Bio-sciences AB, Inc., Uppsala, Sweden). Relative protein abundance was measured using ImageJ software (National Institutes of Health, Bethesda, MD, USA), version 1.8.0.

### 2.7. RT–qPCR

Total RNA was extracted from colon tissues by using TRIzol reagent, and the RNA concentration was measured by using a Nano Photometer P-class (Implen GmbH, Munich, Germany). Then, mRNA was reverse-transcribed into cDNA. The quantitative real-time PCR was performed according to the kit’s instructions (TaKaRa, Osaka, Japan). The relative expression levels of the target genes were analyzed based on the 2^−∆∆Ct^ method. The primer sequences are listed in Supplementary Table S1.

### 2.8. Immunofluorescence (IF) Staining

The colon tissue slices were deparaffinized and then the slices were treated for antigenic repair with sodium citrate antigen repair solution (Beyotime Biotechnology, Shanghai, China). Subsequently, the intestine sections were incubated overnight at 4 °C with primary antibodies, including F4/80^+^ and CD11b^+^. Nuclei were counterstained using 4, 6-diamidino-2-phenylindole (DAPI) (Beijing Solarbio Science & Technology Co., Ltd., Beijing, China). Finally, the intensity of the immunofluorescence staining of specific antibodies was observed using laser scanning confocal microscopy (LSCM) (ECLIPSE Ti2, Nikon, Tokyo, Japan).

### 2.9. Terminal Deoxynucleotidyl Transferase-Mediated dUTP Nick End Labeling (TUNEL) Assay

Apoptosis in the colon tissue was assessed by TUNEL staining, according to the manufacturer’s instructions. Nuclei were stained with DAPI (G8170, Beijing Solarbio Science & Technology Co., Ltd., Beijing, China) and the apoptosis of the intestinal cells was observed under fluorescent microscopy (TCS SPE, Lecia, Wetzlar, Germany) and representative images were obtained.

### 2.10. Statistical Analysis

Data were documented in Excel and analyzed via SPSS (Version 23.0). The results are expressed as the means ± SEMs. One-way ANOVA was used to test the significance of differences, and Duncan’s method was used for comparison between multiple groups; *p* < 0.05 was considered statistically significant. The graphs were prepared using GraphPad Prism (version 9.0, Graph Pad Software Inc., San Diego, CA, USA).

## 3. Results

### 3.1. The Impact of Fecal Microbiota Transplantation from Cats on Colonic Injury in Mice

**Figure 1:**
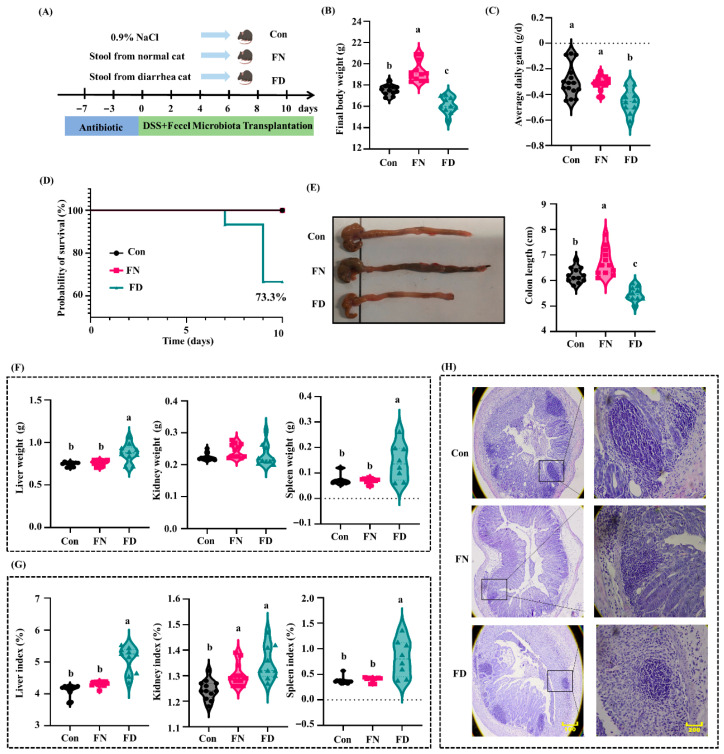
The impact of fecal microbiota transplantation on colonic injury in mice. (**A**) Schematic of infection model. (**B**) Final body weight; (**C**) average daily gain; (**D**) survival curve; (**E**) representative photographs of colon and colonic length; (**F**) organ weight; (**G**) organ index; (**H**) H&E staining of the colon. Data are presented as the mean ± SEMs (n = 12). Different letters indicate significant differences (*p* < 0.05).

Compared with the Con group, the body weight of mice in the FN group was significantly higher, and the body weight of mice in the FD group was significantly lower (Figure 1B). The average daily weight gain of the Con group and the FN group was significantly higher than that of the FD group, and there was no significant difference between the Con group and the FN group (Figure 1C). The survival rate in the FD group was 73.3%, while no fatalities were recorded in either the FN group or the Con group (Figure 1D).

Colonic morphology analysis demonstrated that the colonic length of the FN group was significantly longer than that of the Con group, while that of the Con group was significantly longer than that of the FD group. Additionally, compared with the Con group and the FN group, the contents of the colon and cecum in the FD group mice were significantly reduced (Figure 1E). The liver weights, spleen weights, liver index and spleen index of the FD group were significantly higher than those of Con and FN groups, and the kidney weights showed no significant differences among all groups (Figure 1F,G). Compared with the Con group, the colon of the FD group was characterized by a large number of neutrophils and a small number of lymphocyte infiltration, and the intact intestinal structure was no longer visible. However, the intestinal morphology of the FN group was superior to that of the Con group (Figure 1H). These findings indicate that the microbiota from diarrheic cat feces can exacerbate DSS-induced colitis, while the microbiota from healthy cat feces can ameliorate DSS-induced colitis.

### 3.2. The Impact of FMT on Intestinal Barrier and Inflammation in Mice

**Figure 2:**
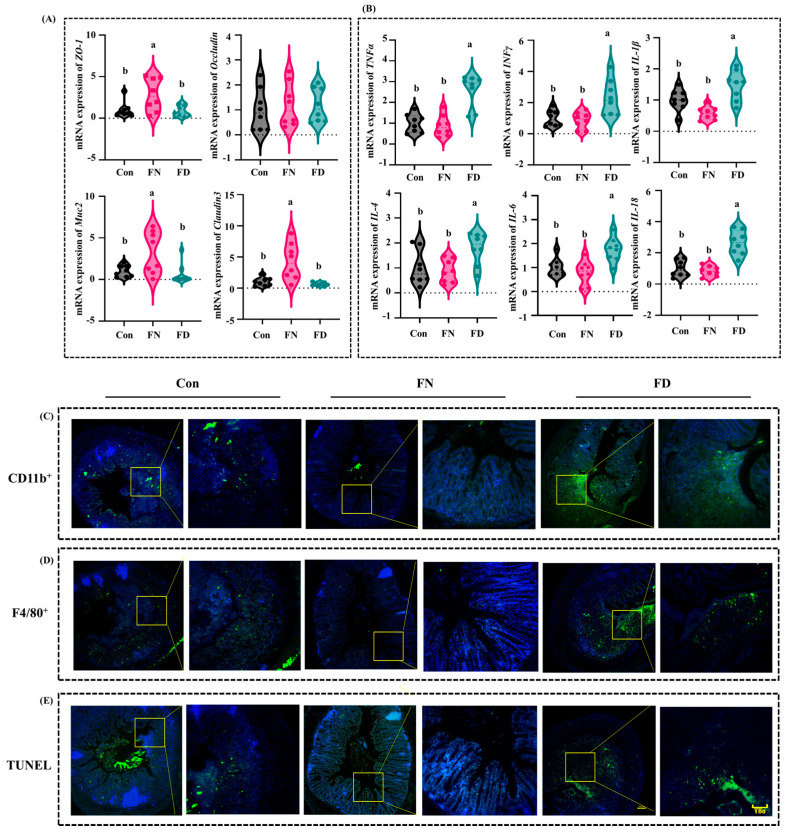
The impact of FMT on intestinal barrier and inflammation in mice. (**A**) Relative mRNA levels of intestinal barrier genes; (**B**) Relative mRNA levels of inflammatory cytokines; (**C**) Representative images of immunofluorescent staining of CD11b^+^ macrophages in the colonic tissues of mice; (**D**) Representative images of immunofluorescent staining of F4/80^+^ macrophages in the colonic tissues of mice; (**E**) Representative images of TUNEL assay of colonic tissues. Data are presented as the mean ± SEMs (n = 8). Different letters indicate significant differences (*p* < 0.05).

The expression levels of the intestinal barrier-related genes ZO-1, Muc2, and claudin-3 were significantly higher in the FN group compared to the Con group and the FD group; there were no significant differences between the Con group and the FD group. The gene expression level of Occludin showed no significant differences among the three groups (Figure 2A). The expression levels of pro-inflammatory factors (TNF-α, IL-4, IFN-γ, IL-1β, IL-6, and IL-18) in the FD group were significantly higher compared to the FN and Con groups (Figure 2B). There were no significant differences between the Con group and the FN group.

CD11b^+^ and F4/80^+^ are two commonly used immune cell markers, which were used to label macrophages of monocyte origin. The number of macrophages in the FD group was significantly higher than the Con group and FN group (Figure 2C,D). TUNEL staining showed that the number of apoptotic cells in the colon of the FD group was higher compared with the Con group and the FN group (Figure 2E). These findings confirm that the microbiota from diarrheic cat feces can exacerbate the inflammatory response in mice with DSS-induced colitis. In contrast, the microbiota from healthy cat feces can mitigate DSS-induced colonic inflammation.

### 3.3. The 16S rDNA Sequencing of Gut Microbiota in Healthy or Diarrheic Cats

**Figure 3:**
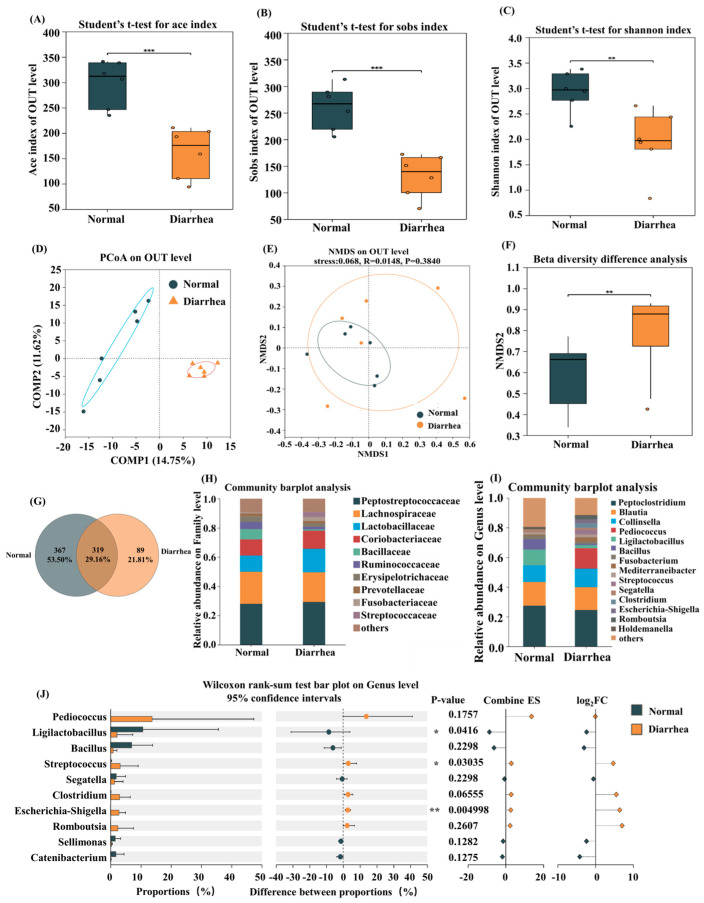
Distinctions of gut microbiota between healthy and diarrheic cats. (**A**) Ace index; (**B**) sobs index; (**C**) Shannon index; (**D**) PCoA; (**E**) NMDS; (**F**) beta diversity; (**G**) Venn diagram; (**H**) relative abundance at the family level; (**I**) relative abundance at the genus level; (**J**) Wilcoxon rank-sum test bar plot at the genus level. Data are presented as the mean ± SEMs (n = 6). * represents a significant difference (*p* < 0.05), ** represents a very significant difference (*p* < 0.01), and *** represents an extremely significant difference (*p* < 0.001).

The ace index, which was used to assess species richness, showed that the ace index of the FN group (healthy cats) was significantly higher than that of the FD group (diarrheic cats) (Figure 3A). The sobs index exhibited a similar trend (Figure 3B). The Shannon index, which measures species diversity, indicated that the FN group had a significantly higher Shannon index than the FD group, suggesting greater species diversity in the healthy group (Figure 3C). Principal Coordinate Analysis (PCoA) revealed a significant difference in the gut microbiota between healthy and diarrheic cats (Figure 3D). Non-metric Multidimensional Scaling (NMDS) results showed that the microbiota from healthy cat feces tended to cluster together, while those from diarrheic cat feces were more dispersed (Figure 3E). Beta diversity analysis indicated that the FN group had significantly lower beta diversity than the FD group (Figure 3F). These findings suggest that although the microbial diversity in the FD group is significantly lower than that in the FN group, the changes in microbial community structure are more pronounced. As shown by the Venn diagram, there are 367 unique OTUs in the FN group, while the FD group has 89 unique OTUs (Figure 3G). At the family and genus levels, the abundance of *Ligilactobacillus* and *Bacillus* was significantly higher in the FN group, while the abundance of *Shigella* was significantly higher in the FD group (Figure 3H–J).

### 3.4. Isolation of Pathogenic from the Feces of Cats

**Figure 4:**
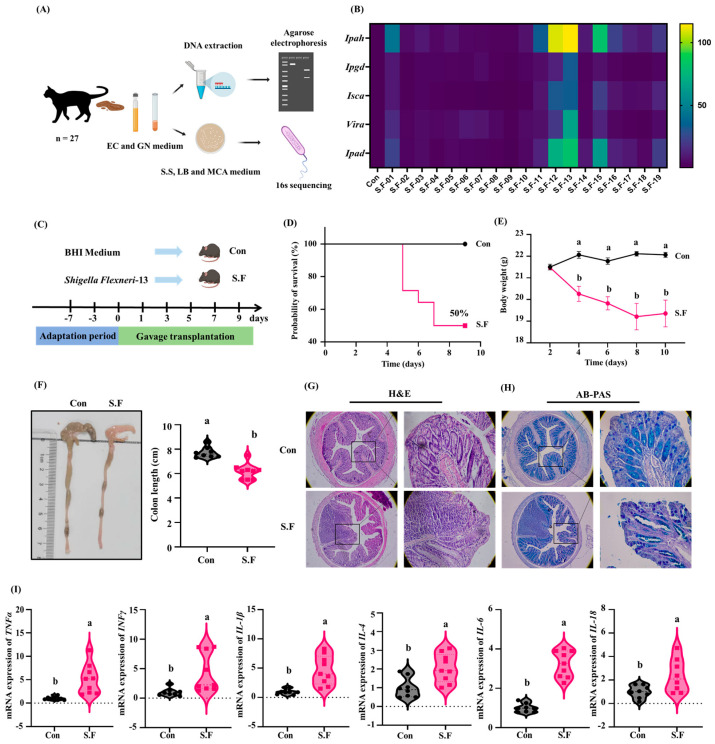
Isolation and identification of pathogenic bacteria in feces of cats with diarrhea. (**A**) Schematic progress of bacterial isolation; (**B**) relative mRNA levels of virulence factor-related genes of *Shigella flexneri*; (**C**) schematic of infection model. (**D**) Survival curve; (**E**) body weight curve; (**F**) representative photographs of colon and colonic length; (**G**) H&E staining of the colon; (**H**) AB-PAS staining of the colon. (**I**) Relative mRNA levels of inflammatory cytokines; data are presented as the mean ± SEMs (n ≥ 12). Different letters indicate significant differences (*p* < 0.05).

Escherichia coli Broth (EC) and Gentamicin Neomycin medium (GN) were used to enrich and isolate bacteria from the feces, and 19 strains of *Shigella flexneri* were isolated from the feces of 27 diarrheic cats (Figure 4A). Among the *Shigella* strains, we found that the virulence gene expression level of *Shigella flexneri 13* was significantly higher than that of the other strains (Figure 4B) and further study was conducted to evaluate its deleterious effect on the colonic barrier in mice.

Six-week-old C57BL/6J mice treated with a cocktail of four antibiotics were randomly divided into two groups, which were orally administered with normal saline (Con) or *Shigella flexneri 13* (2 × 10^7^ CFU/mL, 200 μL/d) (S.F), respectively (Figure 4C). The survival rate of mice infected with *S. flexneri* was 50% (Figure 4D). As is shown, mice in the S.F group had significantly lower body weight compared to the Con group (Figure 4E). Morphological analysis revealed that the colonic length in the S.F. group was significantly shorter compared to the Con group. Additionally, in the S.F group, the contents of the colon and cecum were not distended (Figure 4F). Through histological examination with H&E and AB-PAS staining techniques, it was observed that the mice in the S.F group exhibited immune cell infiltration and a decreased level of mucin content (Figure 4G,H). The expression levels of pro-inflammatory cytokines (TNF-α, IL-1β, IFN-γ, IL-4, IL-6, and IL-18) in the S.F group were significantly higher compared to the Con group (Figure 4I).

### 3.5. Weizmannia coagulans Long45 Alleviates Shigella flexneri-Induced Colitis in Mice

**Figure 5:**
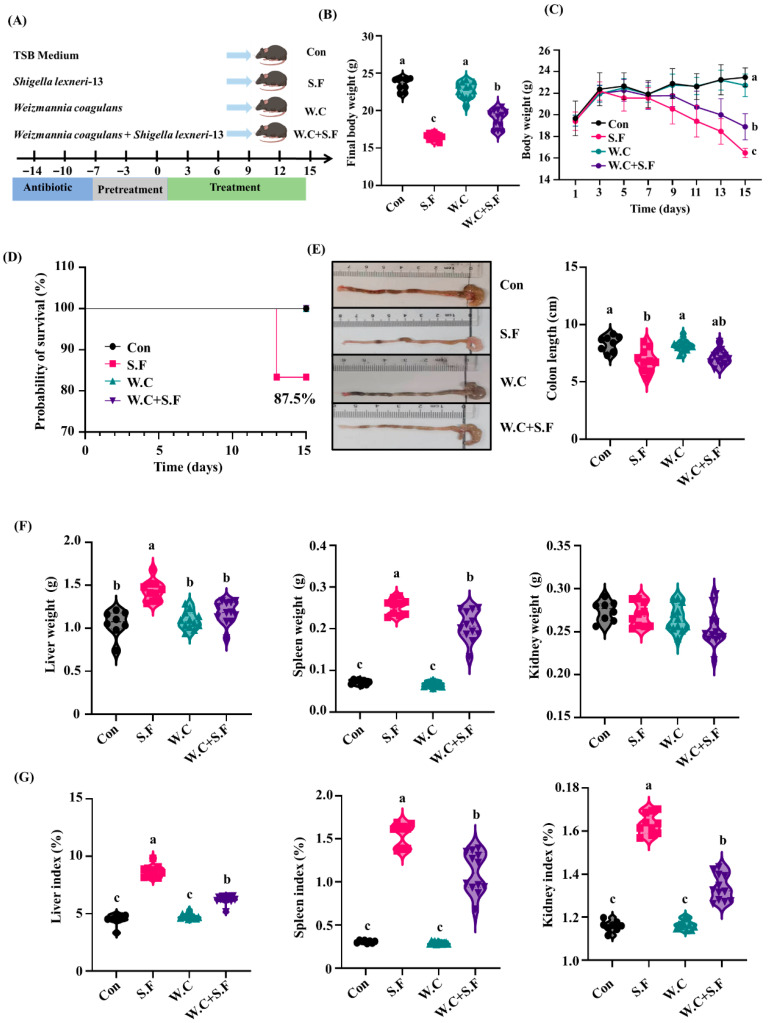
*Weizmannia coagulans* alleviates *Shigella*-induced weight loss and organ index elevation in mice. (**A**) Schematic of infection model. (**B**) Final body weight; (**C**) Body weight curve; (**D**) Survival curve; (**E**) Representative photographs of colon and colonic length; (**F**) Organ weight; (**G**) Organ index; data are presented as the mean ± SEMs (n = 12). Different letters indicate significant differences (*p* < 0.05).

In our recent study, we isolated several strains of probiotics from the fecal samples of 10 healthy cats. A comprehensive phylogenetic analysis showed that *Weizmannia coagulans Long45* had no adverse effects on the health of mice (Figure S1) and might confer a beneficial effect on intestinal health. To test this hypothesis, six-week-old C57BL/6J mice treated with a cocktail of four antibiotics were randomly divided into four groups. From days 1 to 7, mice in the Con and W.C groups were administered Tryptic Soy Broth (TSB) medium via gavage and mice in the S.F and W.C+ S.F groups were given *Shigella flexneri Long13*. From days 8 to 23, mice in the Con and S.F groups continued to receive TSB medium, whereas mice in the W.C and W.C+ S.F groups were administered *Weizmannia coagulans Long45* (Figure 5A).

Compared with the Con group, *Shigella flexneri 13* infection led to a significant decrease in body weight in mice, whereas the administration of *Weizmannia coagulans Long45* effectively mitigated this effect (Figure 5B,C). Notably, the survival rate of mice in the S.F group was 87.5%, while no fatalities were recorded in the other three groups (Figure 5D). Morphology analysis showed that *Shigella flexneri 13* infection caused a significant shortening of the colon length and a marked reduction in the cecal and colonic contents in the mice, compared with the Con group. However, following the administration of *Weizmannia coagulans* Long45, a trend towards alleviation of these changes was observed (Figure 5E). Additionally, the liver weights, liver indices, spleen weights, spleen indices and kidney indices of mice in the S.F group were significantly higher than those in the Con group, which were reversed by administration of *Weizmannia coagulans* Long45 (Figure 5F,G).

### 3.6. Weizmannia coagulans Alleviates Shigella flexneri-Induced Colonic Barrier Damage

**Figure 6:**
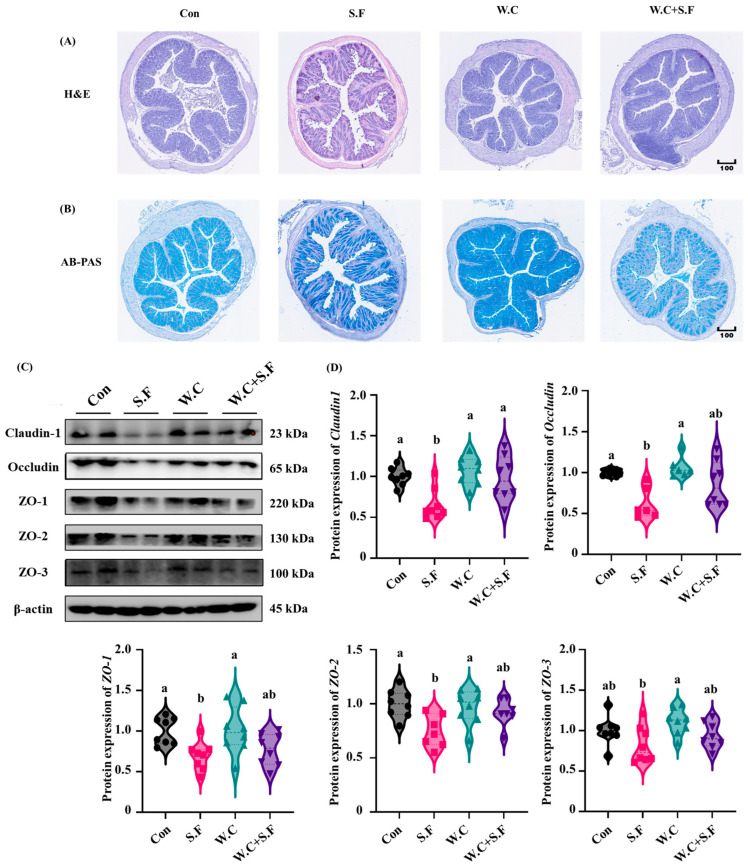
*Weizmannia coagulans* alleviates *Shigella*-induced colonic barrier damage. (**A**) H&E staining of the colon; (**B**) AB-PAS staining of the colon. (**C**,**D**) Western blot bands and abundance analysis of proteins involved in intestinal barrier in the colon. Data are presented as the mean ± SEMs (n = 8). Different letters indicate significant differences (*p* < 0.05).

Histological examination of the colon via H&E and AB-PAS staining revealed that the colonic epithelial cells in the S.F group exhibited shedding and a reduction in mucin content compared to the Con group. However, *Weizmannia coagulans* Long45 supplementation significantly mitigated these adverse effects (Figure 6A,B). and exerted a protective effect on the colonic barrier. In comparison to the Con group, the S.F group demonstrated a statistically significant decrease in the protein abundance levels of Claudin-1, Occludin, ZO-1, ZO-2 and ZO-3. Following supplementation with *Weizmannia coagulans* Long45, the decline in the abundance of colonic barrier proteins, induced by *Shigella flexneri*, was substantially mitigated (Figure 6C,D). In summary, *Weizmannia coagulans* Long45 exerts a mitigating effect on the colonic barrier damage induced by *Shigella flexneri*. This effect is achieved through the regulation of tight junction protein abundance.

### 3.7. Weizmannia coagulans Alleviates Oxidative Stress and Apoptosis in Colonic Cells Induced by Shigella flexneri

**Figure 7:**
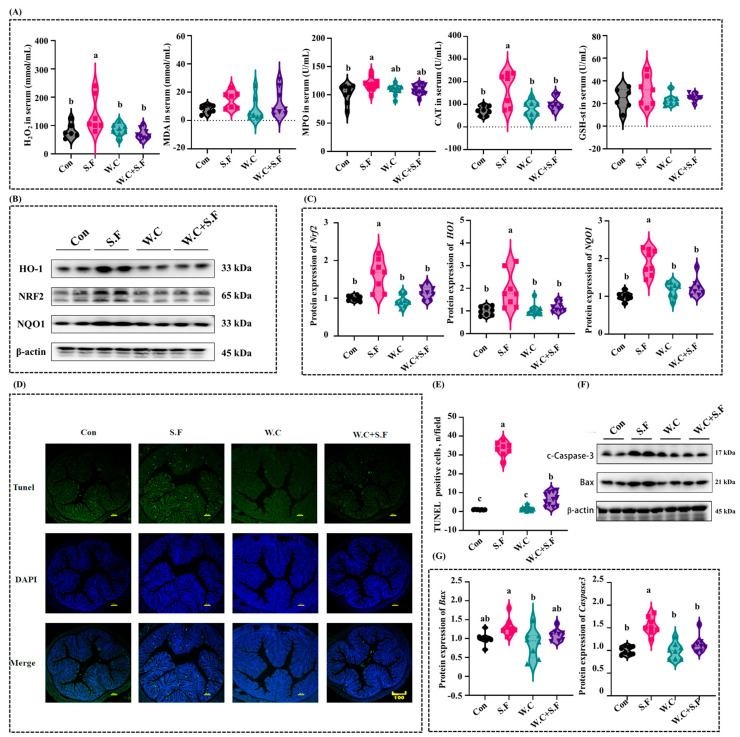
*Weizmannia coagulans* alleviates oxidative stress and apoptosis in colonic cells. (**A**) The levels of H_2_O_2_, MDA, MPO, CAT, and GSH-st in the serum were determined by using commercial kits; (**B**) Western blot analysis of NQO1, HO-1 and Nrf2 in the colon and (**C**) Statistical analysis of protein abundance; (**D**,**E**) Representative images of TUNEL assay of colonic tissues; (**F**) Western blot bands and abundance analysis of proteins involved in apoptosis in colon and (**G**) Statistical analysis of protein abundance. Data are presented as the mean ± SEMs (n = 8). Different letters indicate significant differences (*p* < 0.05).

In comparison to the Con group, following infection of mice with *Shigella flexneri 13*, there was a statistically significant elevation in the serum concentrations of hydrogen peroxide (H_2_O_2_), myeloperoxidase (MPO), and catalase (CAT). Upon supplementation with *Weizmannia coagulans Long45*, the increase in H_2_O_2_, MPO, and CAT levels induced by *Shigella flexneri* infection was substantially mitigated. There were no statistically significant differences in the serum levels of malondialdehyde (MDA) and reduced glutathione S-transferase (GSH-ST) among all groups (Figure 7A). Compared with the Con group, the abundance of proteins such as HO-1, Nrf2 and NQO1 significantly increased in mice infected with *Shigella flexneri Long13*, and this increase was significantly attenuated by *Weizmannia coagulans* supplementation (Figure 7B,C). Immunofluorescence staining revealed that the number of apoptotic cells in the colons of mice significantly increased following *Shigella flexneri* infection. However, this increase was significantly attenuated when the mice were supplemented with *Weizmannia coagulans* (Figure 7D,E). When compared to the Con group, there was a notable increase in the abundance of proteins like cleaved caspase-3 in mice infected with *Shigella flexneri*. However, this increase was significantly mitigated by the supplementation of *Weizmannia coagulans*. The expression pattern of Bax was consistent with c-Caspase-3. Only the S.F group showed significantly higher Bax expression than the W.C group, with no significant differences observed in other groups (Figure 7F,G).

### 3.8. Weizmannia coagulans Supplementation Inactivates TLR2/TLR4-MyD88-NF-κB Signaling

**Figure 8:**
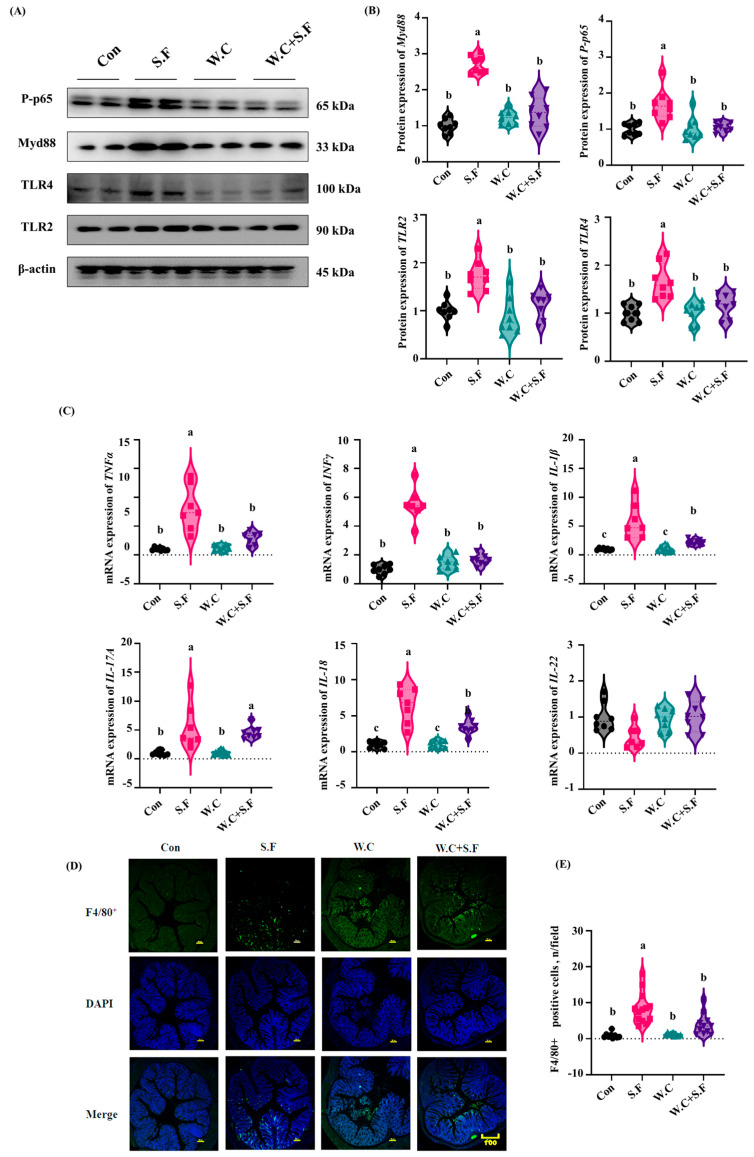
*Weizmannia coagulans* regulates the TLR2/TLR4-MyD88-NF-κB pathway to control inflammation. (**A**,**B**) Western blot bands and abundance analysis of proteins involved in inflammation-related proteins in colon; (**C**) Relative mRNA levels of inflammatory cytokines; (**D**,**E**) Representative images of immunofluorescent staining of F4/80^+^ macrophages in the colonic tissues of mice. Data are presented as the mean ± SEMs (n = 8). Different letters indicate significant differences (*p* < 0.05).

In comparison to the Con group, mice infected with *Shigella flexneri Long13* exhibited a statistically significant elevation in the protein abundance of P-p65, Myd88, TLR2 and TLR4, and this was reversed by *Weizmannia coagulans Long45* supplementation (Figure 8A,B). In addition, the expression levels of pro-inflammatory genes (TNF-α, IFN-γ, IL-1β, IL-17A, and IL-18) were significantly increased in the colons of *Shigella*-infected mice, and these effects were reversed by *Weizmannia coagulans* administration (Figure 8C). Immunofluorescence staining demonstrated that the quantity of macrophages in the colons of mice exhibited a statistically significant elevation in the *Shigella*-infected group compared to the Con group. However, these alterations were markedly attenuated following supplementation with *Weizmannia coagulans* (Figure 8D,E).

## 4. Discussion

In the present study, we found that feline-derived *Shigella flexneri 13* infection can induce colitis, as demonstrated by the colonic injury, inflammation response and immune cell invasion in the mice. Importantly, the deleterious effects of *Shigella flexneri* were significantly reversed by *Weizmannia coagulans* Long45 supplementation, indicating a potential therapeutic treatment option for intestinal health issues.

*Shigella flexneri* is the most common pathogen causing intestinal disease in both developing and developed countries. It can survive in harsh environments and cause bacterial dysentery in humans and certain primates, leading to symptoms such as fever, abdominal pain, diarrhea, dehydration, nausea and vomiting, but it usually does not demonstrate significant pathogenicity in non-primate animals [22,23]. Despite significant progress related to food-derived *Shigella flexneri* and its deleterious effects on human health, it is still unknown whether feline-derived *Shigella flexneri* contributes to the intestinal health of humans and animals, considering the close relationship between pet owners and their companion animals, such as dogs and cats. To test our hypothesis, the fecal microbiota from healthy or diseased cats diagnosed with bacterial-related colitis were selected, and their fecal microbiota was prepared according to a previously described protocol [24]. All the animals were inoculated with vaccines and no parasites were observed in the feces, therefore eliminating the potential causes of diarrhea associated with viral or parasitic infections. Then, we transplanted the fecal microbiota (10^11^ cfu/mL, 200 μL) from diarrheic or healthy cats, respectively, into healthy mice pretreated with antibiotic cocktails to exclude endogenous bacteria interference and assist the colonization of the fecal microbiota. The results revealed that the transplantation of microbiota from diarrheic cats exacerbated colon injuries, as demonstrated by body weight loss, decreased colon length shorting, mortality rate, increased immune cell infiltration, enhanced mRNA levels of inflammatory cytokines (TNF-α, IL-4, IFN-γ, IL-1β, IL-6, and IL-18), and increased cell apoptosis. In contrast, the transplantation of microbiota from healthy cats was associated with attenuated colon length shortening, increased body weight, increased mRNA level of tight junction genes (ZO-1 and Claudin-1), Muc2, and decreased apoptosis and immune cell infiltration. These data clearly demonstrate that the fecal microbiota of diseased cats is critical for intestinal barrier breakdown and colitis, while the fecal microbiota of healthy cats is involved in intestinal injury repair [25,26,27].

To gain a deeper understanding of the alterations in the gut microbiota of cats experiencing diarrhea, we compared the 16S rDNA sequences of the fecal microbiota and found that at the genus level, there was a statistically significant elevation in the abundance of *Shigella,* along with a marked and statistically significant decline in the abundance of *Bacillus* within the feces of diarrheic cats. Using the classic bacterial isolation method, we isolated 19 strains of *Shigella* from the feces of cats with diarrhea. Among these strains, we found that *Shigella flexneri 13* was the most virulent and infection with this strain led to the colitis-related signs, which were similar to the phenotypes exhibited in the mice that received fecal microbiota transplantation from diarrheic cats. These results indicate the existence of feline-derived *Shigella*, which may be a risk factor for both humans and companion animals [28].

To test for the beneficial effect of probiotic supplementation on feline-derived *Shigella*-induced colitis, mice treated with *Shigella flexneri 13* were administered with probiotic *Weizmannia coagulans* Long45. Previous studies have found that *Weizmannia coagulans,* a Gram-positive and facultative anaerobic bacterium, possesses the remarkable ability to form highly resilient spores under unfavorable conditions [29,30], with ability to regulate the host’s immune response, reduce intestinal inflammation, and enhance the body’s resistance to pathogens [31,32]. However, there is currently no evidence showing that *Weizmannia coagulans* can prevent feline-derived *Shigella*-induced barrier dysfunction. In our study, we found that *Shigella flexneri*-induced phenotypes, including body weight alteration, mortality rate, decrease in tight junction proteins (Claudin-1, Occludin, ZO-1, ZO-2 and ZO-3) and increase in cytokines (TNF-α, IL-4, IFN-γ, IL-1β, IL-6, and IL-18), were significantly improved by *Weizmannia coagulans* supplementation. In addition, the increased serum levels of H_2_O_2_ and CAT triggered by *Shigella flexneri* infection were significantly mitigated by *Weizmannia coagulans* administration, indicating a regulatory effect on the oxidative and redox balance under this condition. Also *Shigella* infection induced a significant compensatory upregulation of the Nrf2 antioxidant pathway, characterized by elevated Nrf2, HO-1, and NQO1 expression, as a host defense mechanism to counteract pathogen-induced oxidative stress. *W. coagulans* treatment effectively mitigated *Shigella*-induced intestinal inflammation and ROS production, which in turn normalized Nrf2 pathway activation to baseline levels. This normalization directly reflects the reduction in oxidative stress and tissue damage, confirming a protective effect of *W. coagulans* against *Shigella*-mediated intestinal injuries.

The activation of toll-like receptor (TLR)-mediated signal transduction and downstream transcriptional factor NF-κB is critical for Gram-negative bacterial infections, leading to the release of various cytokines [33,34]. To explore the involvement of NF-κB signaling in *Shigella flexneri*-induced inflammation and a potential protective effect of *Weizmannia coagulans*, we determined the protein levels of TLR2, TLR4, and Myd88, and the phosphorylation of NF-κB, as well as pro-inflammatory cytokines. As expected, *Shigella flexneri* induced the activation of TLR-Myd88-NF-κB signaling, as well increased cytokines, such as TNF-α, IL-1β, and IL-6, which were largely abolished by probiotic treatment. These results, along with the immunofluorescence staining results, indicate a regulatory effect of probiotics on immune cell infiltration and cytokine synthesis. It has been reported that the proliferation of *Shigella* within the intestinal tract is accompanied by the synthesis of lipopolysaccharide, which can be sensed by intestinal epithelial cells [35,36,37]. Release of lipopolysaccharide leads to subsequent activation of NF-κB signaling in a TLR-dependent manner, resulting in increased levels of pro-inflammatory cytokines and the recruitment of macrophages [33,37]. *Weizmannia coagulans* supplementation inactivates TLR-Myd88-NF-κB signaling to restore intestinal homeostasis.

## 5. Conclusions

In summary, using fecal microbiota transplantation technology, we found that the transplantation of fecal microbiota from diseased cats exacerbates colitis injury and intestinal barrier disruption. A comparative study of the 16S rDNA sequencing revealed that *Shigella* is a potential pathogen related to deleterious effects. This hypothesis was confirmed by the isolation of *Shigella flexneri* and phenotype simulation. Importantly, we demonstrated that *Weizmannia coagulans* Long45 alleviates pathological alteration and colonic barrier dysfunction by modulating the MyD88-NF-κB and Nrf2 signaling pathways. Further studies are required to explore whether feline-derived *Shigella flexneri* can be transmitted to humans and contribute to the intestinal dysfunction of companion animal owners.

## Data Availability

All data needed to assess the conclusions in this study are provided in the manuscript and/or the Supplementary Materials. The data used in this study are available from the science data bank, and can be accessed via https://doi.org/10.57760/sciencedb.31457.

## Supplementary Materials

The following supporting information can be downloaded at: https://www.mdpi.com/article/10.3390/nu18101486/s1, Figure S1: *Weizmannia coagulans* Long45 evolutionary tree; Table S1: Primer sequence.

## Abbreviations

AB-PAS	Alcian blue/periodic acid–Schiff
CAT	Catalase
DSS	Dextran sulphate sodium
EC	Escherichia coli broth
FMT	Fecal microbiota transplantation
GSH-ST	Glutathione S-transferase
GN	Gentamicin neomycin medium
H&E	Hematoxylin–eosin
H_2_O_2_	Hydrogen peroxide
IL	Interleukin
IFN-γ	Interferon-gamma
MDA	Malondialdehyde
MPO	Myeloperoxidase
NMDS	Non-metric multidimensional scaling
OUT	Operational taxonomic unit
PCoA	Principal coordinate analysis
TLR	Toll-like receptor
TNF-α	Tumor necrosis factor-alpha
TSB	Tryptic soy broth medium
