# Supplementary material for: Weizmannia coagulans Long45 Supplementation Prevents Feline-Derived Shigella flexneri 13-Induced Colitis in Mice by Regulation the Nrf2 and NF-κB Signaling Pathways
Source: Nutrients. 2026 May 7;18(10):1486. doi: 10.3390/nu18101486 (PMC13209405; doi:10.3390/nu18101486)
Supplement: Supplementary file 1 [file nutrients-18-01486-s001.zip › nutrients-4227868-supplementary.pdf]

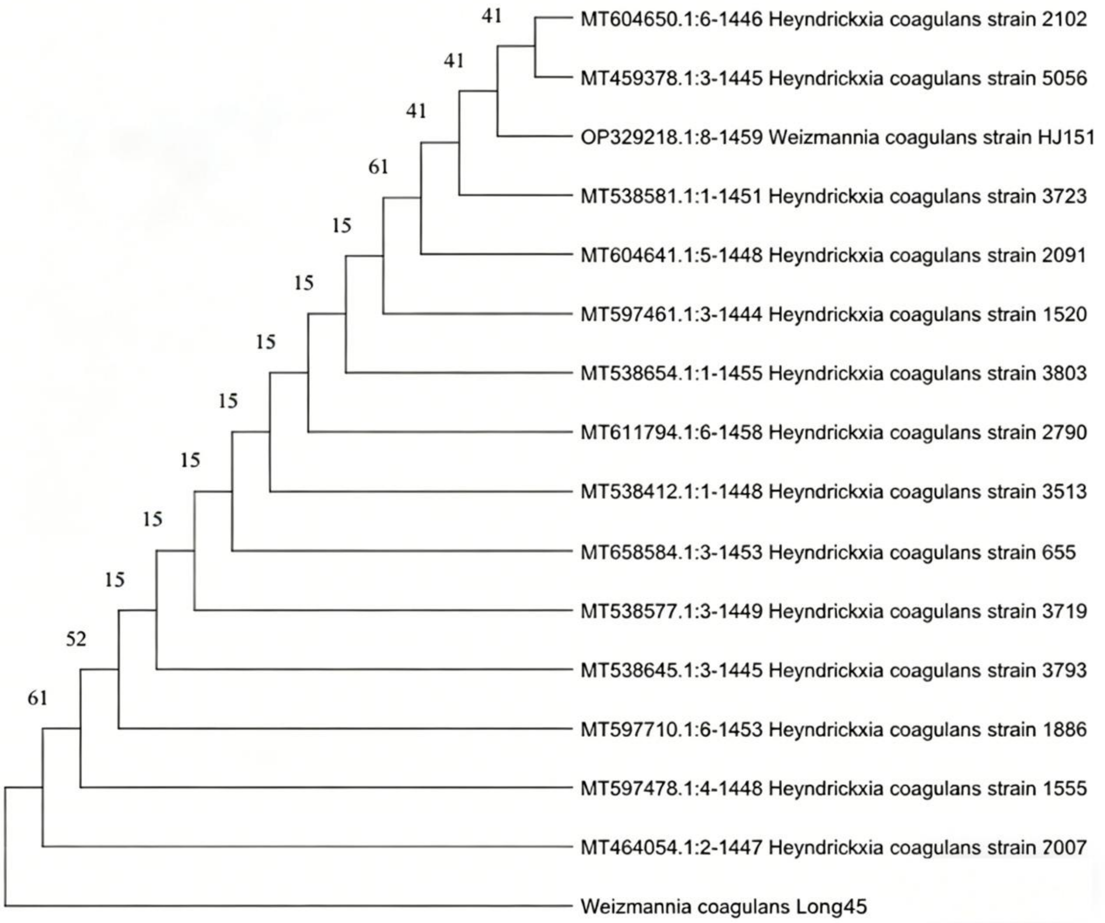

Supplementary Figure S1 *Weizmannia coagulans* Long45 evolutionary tree.

Supplementary Table S1

Item	Gene		Sequence（5'→3'）
1	Occludin	F	CTTCGACTCGCTGCTGAATC
		R	ACCTCATCGTCTTCCATGCA
2	ZO-1	F	GCCCACCAAGATCGTCTACT
		R	GTAGTCCTTGCGGTCGTAGG
3	Muc1	F	ATGCCCTTGCGTCCATAACA
		R	AGGAGCAGTGTCCGTCAAAG
4	Muc2	F	TGGAACCACGATGACAGCCT

		R	TTCCCGAATTCCATGGGTGT
7	IFN-γ	F	GTCAACAACCCACAGGTCCA
		R	ACTCCTTTTCCGCTTCCTGA
8	TNFα	F	CCACCACGCTCTTCTGTCTA
		R	GGTCTGGGCCATAGAACTGA
9	IL-4	F	ACTTGAGAGAGATCATCGGCA
		R	AGCTCCATGAGAACACTAGAGTT
10	IL-6	F	CTGCAAGAGACTTCCATCCAG
		R	AGTGGTATAGACAGGTCTGTTGG
11	IL-17	F	ACCTGGAACTGAATGCCTGA
		R	GTCCCTCGATGTGGCTACTT
12	IL-18	F	CCCAATGAGTAGGCTGGAGA
		R	TCTGGACCCATTCCTTCTTG

6
